# Measuring cyber wisdom: preliminary validation of a new four-component measure

**DOI:** 10.1007/s10639-023-11953-9

**Published:** 2023-07-04

**Authors:** Tom Harrison, Gianfranco Polizzi, Shane McLoughlin, Francisco Moller

**Affiliations:** 1 University of Birmingham, Birmingham, UK; 2 University of Liverpool, Liverpool, UK

**Keywords:** Cyber-wisdom, Measures, Digital Citizenship Education, Adolescents

## Abstract

Cyber-wisdom is the ability to know and do the right thing at the right time, when using digital technologies, and is a concept that is gaining attention from educators. Whilst the theory and practice of cyber-wisdom education is established, to date there has been no attempt to investigate how the virtue of cyber-wisdom might be measured. This is a lacuna as it limits future research in the area, including, in particular, proximal evaluations of cyber-wisdom interventions. This article introduces a new four-component measure of cyber-wisdom, which is relevant to how the virtue may be cultivated in practice via formal education and the teaching of what is generally referred to as digital citizenship education. The measure was piloted with 1,331 13–16 year-olds. The findings provide initial evidence that cyber-wisdom literacy, reasoning, reflection, and motivation can be measured. This study provides preliminary validation of cyber-wisdom sub-measures that might be used in evaluations of educational interventions that seek to help children and adolescents live with wisdom in the digital age.

## Background

Children and adolescents are at the forefront of using digital technologies. This means they often experience both the opportunities and risks that digital technologies present far earlier than older users. On the one hand, children and adolescents enjoy opportunities for learning, socialisation, leisure, employment, and participation. On the other hand, they are also among the first users to experience issues of online abuse (e.g. cyberbullying, trolling), invasion of privacy, misinformation and security, to name a few (Livingstone et al., 2018). As children and adolescents grow up, they are increasingly presented with situations that require them to navigate both the opportunities and risks that these digital technologies present. It is therefore perhaps not surprising that according to a recent Organisation for Economic Cooperation and Development (OECD) report (Burns & Gottschalk, 2020, p. 46), promotion of digital citizenship education is largely perceived across countries in the world, including the UK, as the most important global challenge of the digital age.

Research that is primarily theoretical in nature has made the case for prioritising the education of cyber-wisdom in schooling (see, for example, Harrison et al., 2022a) as a form of digital citizenship education (teaching pupils how to become responsible users of digital technologies with a view to participating in society). It is argued that if children and adolescents possess cyber-wisdom they are more likely to grab opportunities and less likely to succumb to the risks of living large parts of their lives online. Cyber-wisdom may be defined as the ability to know and do the right thing at the right time, when using digital technologies (Harrison, 2016). Meanwhile, cyber-wisdom education refers to the teaching and learning of the four components of cyber-wisdom (explained in detail below). While schools often focus on the teaching of digital literacy – the skills and knowledge that pupils need in order to use digital technologies both functionally and critically (see, for example, Polizzi & Harrison, 2020) – we argue that if we are to expect children and young people to act responsibly as citizens in the digital age, then digital citizenship education needs to be promoted more robustly in ways that incorporate cyber-wisdom education.

In the digital world where many children spend much of their lives, the consequences of their actions are often hard to predict and rules hard to enforce. This makes educational approaches to digital citizenship education that are informed by either deontological (rules-based) or utilitarian (consequence-based) moral theories impoverished. In contrast, approaches that prioritise the education of character qualities that help users minimise the risks and maximise the opportunities of being online are much needed by teachers and other educators. It is therefore not surprising that leaders in the digital education field, like Common Sense Media^1^, are increasingly placing emphasis on character education for the digital age. Hampering these efforts is a lack of instruments and scales to measure, and evaluate interventions that focus on, the character qualities required to flourish online. It is this gap that the current article tackles. In this article, we introduce a new four-component measure for cyber-wisdom and promising evidence about its validity as a measure that can be used to evaluate new character-based approaches to digital citizenship education interventions. The article deepens and extends the analysis of measures used in a feasibility study of an intervention designed to educate the components of cyber-wisdom (Harrison et al., 2022b). It also builds both on the theoretical model of cyber-wisdom, and how this can be cultivated in practice via formal education, that was published in the Journal of Ethics and Information Technology (Polizzi et al., 2022c) and on empirical research that found that cyber-wisdom is valued by both parents and adolescents that was published in the Journal of Education and Information Technologies (Harrison et al., 2022a).

After introducing the context for the article and the four-component measure, we explain the methodology we used to pilot the measure. Following the findings from this pilot psychometric study, in the final section we discuss the implications of this research and provide insights that could help inform future research and practice in the field of character education, digital citizenship education, and education and information technologies more widely. This study is particularly relevant to those who may be tasked with developing, implementing, and evaluating programmes of digital citizenship education in schools that are designed to contribute to pupils’ online flourishing. The findings from this study aim to provide a potential road map for both researchers and practitioners concerned with the education of the character virtues that adolescents need in order to thrive in the digital age. Given that this was the first known attempt to define and then measure four components of cyber-wisdom, the modest goal for the article is to lay the foundations for future longitudinal research on cyber-wisdom education that may be carried out on a larger scale and utilising more advanced evaluative research methodology.

### A character based approach to digital citizenship education

To flourish in the digital age, children and adolescents need to possess and deploy character qualities that inform how they use technology to interact with others and participate in society. This means that digital citizenship education, which teaches students how to use technology responsibly, should overlap with character education. It is surprising that this form of education has a marginal place in school curricula (Harrison et al., 2022a, b), despite the fact that organisations like Common Sense Media, the Council of Europe, and the Jubilee Centre for Character and Virtues have published resources and frameworks that prioritise character development (Jubilee Centre, 2022). In the UK, the Education for a Connected World framework offers guidance on the skills and knowledge children should gain in the classroom. Many schools teach digital citizenship through assemblies, PSHE, citizenship, and computing classes, as well as through communication and advice to parents. We argue that a more comprehensive approach to digital citizenship education is needed, one that is grounded in neo-Aristotelian character education and virtue ethics (Harrison, 2021a, b, 2022a, b).

In recent years, the case for character-based approaches to digital citizenship education has been considerably advanced. In part, this is due to alternative approaches that are primarily underpinned by deontological (rules-based) or utilitarian (consequence -based) moral theories, which have been found to be impoverished. Such approaches are based respectively on imposing rules or restrictions and on encouraging students to be mindful of the consequences of their own online actions. Many schools, for example, seek to ban or restrict mobile phone use during and/or in-between classes, with teachers instructing pupils to respect rules of moral conduct (Humble-Thaden, 2011; Selwyn & Aagaard, 2021). For example, the term *netiquette* is widely used in schools, which is an example of how deontology legitimises the use of rules and norms to dictate what may (or not) be considered appropriate behaviour online. At the same time, utilitarian or consequentialist strategies are based on encouraging children to reflect on the possible repercussions of their online behaviour. This is why schools might show students films about the effects of cyberbullying on adolescents’ mental or physical wellbeing, or about the consequences of sexting (e.g. Morgan, 2013).

While these approaches are important and should be part of a more comprehensive approach to digital citizenship education (see Harrison et al., 2022a), what is unclear is whether they are sufficient to prepare children and adolescents to use digital technologies responsibly (Vallor, 2016; Harrison, 2021a). What is more, research conducted in 2021 by the Jubilee Centre revealed that both parents and adolescents aged 13–16 in the UK prioritised virtue-based over deontological or consequentialist reasoning to justify, respectively, their parental mediation strategies and use of the Internet (Harrison and Polizzi, 2021b). More specifically, the Jubilee Centre found that the explanations that most adolescents provided in support of undertaking morally engaged reactions to an abusive post online (e.g. ‘send a nice message to the person insulted to check how they feel’) were virtue-based (68%) (e.g. ‘because it is the kind/thoughtful thing to do’) as distinct from utilitarian (21%) (e.g. ‘because the same thing might happen to me’), or deontological (11%) (e.g. ‘because of the rules of the social media company’). More importantly, when participants were presented with a list of virtues (including, for example, compassion, honesty and resilience), most adolescents reported wisdom to be the virtue that they wanted their friends to show the most on social media, with 38% choosing this as one of their top two desired qualities. Similarly, wisdom was also reported as the virtue that parents most wanted their children to show online, with 56% choosing this as one of their top two qualities.

### Conceptualising the components of Cyber-Wisdom

Cyber-wisdom – that is, doing the right thing at the right time when online, particularly when no-one is watching – stems from the Aristotelian concept of *phronesis* in ways that apply to the online world (Harrison, 2016). Like the term *phronesis*, which is often translated as practical wisdom, the concept of cyber-wisdom presents features that need to be attuned to the demands of our contemporary societies and, in the case of cyber-wisdom, to the digital age in which we live. It builds on considerable interest and research on the virtue of wisdom in recent philosophical and psychological scholarship (including, notably, Schwartz and Sharpe, 2010; Kristjánsson, 2015; Darnell et al., 2019;). Of particular significance is an article by Grossmann et al. (2020), which seeks to develop a unified understanding of wisdom for the contemporary age and is considered extremely influential in the current literature on wisdom.

Cyber-wisdom, a construct that enables children and adolescents to navigate online risks and opportunities, should be viewed as a complex, multi-component meta-virtue (Polizzi & Harrison, 2020). As classified by the Jubilee Centre (2022), the virtues involved include moral (e.g. compassion), civic (e.g. supporting social justice), intellectual (e.g. independent thought), and performance virtues (e.g. resilience). Cyber-wisdom coordinates these virtues in online situations. Like *phronesis*, but unlike the intellectual virtue of *sophia*, which refers to theoretical wisdom, cyber-wisdom is concerned with the practical application of moral judgments to enhance online behaviour. It is a quality that is refined through experience, experimentation, and reflection on action.

The cyber-wisdom measure that is central to cyber-wisdom education and is reported on here draws on a four-component understanding of cyber-wisdom. In terms of their theoretical and educational properties, these components build on the Jubilee Centre’s research on character education, virtue literacy, and *phronesis.* The components are also grounded in neo-Aristotelian virtue ethics and closely related to three prominent existing models of wisdom (Ardelt, 2004; Darnell et al., 2019; Grossmann et al., 2020). A comprehensive overview of how the four components of cyber-wisdom education have been developed can be viewed in an extended article written by Polizzi and Harrison (2022). The argument offered in the article was that the four components are essential for navigating online risks and opportunities, and necessary for flourishing online. The four components of cyber-wisdom, which have implications for how they may be educated in practice, are: cyber-wisdom literacy; cyber-wisdom reasoning; cyber-wisdom self-reflection; and cyber-wisdom motivation. Unlike the models of wisdom on which cyber-wisdom builds, these components account for the specificity of the digital age and are both conceptual and practical. Each of the components are briefly explained below.

### Cyber-wisdom literacy

Cyber-wisdom literacy is an understanding of virtues such as honesty and compassion in relation to digital technologies. This concept aligns with moral psychologist Ardelt’s (2014) cognition component in her model of wisdom, and with Darnell et al.’s (2019) and Kristjánsson et al.’s (2021) notion of virtue literacy in their model of *phronesis*. These models all emphasise the need for understanding the virtues that apply to different situations. Cyber-wisdom literacy, however, is specifically focused on the digital age and its possibilities and challenges. Therefore, it involves not only understanding the appropriate virtues for online contexts, but also how to take advantage of online opportunities while avoiding online risks. For instance, practising cyber-wisdom literacy could involve accessing online information with virtuous curiosity, while also avoiding the spread of online misinformation by sharing content honestly. Cyber-wisdom literacy can be taught through the use of narratives and stories that encourage students to develop an understanding of virtues in the online world. This method builds on the Jubilee Centre’s (2022) approach to teaching virtue literacy in the classroom, and on the well-known benefits of using narratives and stories for teaching moral character (e.g. Arthur et al., 2014). As such, cyber-wisdom literacy could be integrated into digital literacy education.

### Cyber-wisdom reasoning

Cyber-wisdom reasoning is the ability to prioritise virtues in the context of using digital technologies, especially when they conflict. This component draws on Grossman et al.’s (2020) ‘perspectival meta-cognition’ in their model of wisdom, and on Darnell et al.’s (2019) and Kristjánsson et al.’s (2021) integrative function of *phronesis* as an evaluative approach to dealing with moral dilemmas. Cyber-wisdom reasoning takes into account the ways in which the Internet can exacerbate moral dilemmas, such as the decision to access free online information versus respecting copyright laws, or how to respond to online abuse. Therefore, users need to rely on their experience of using digital technologies to navigate these dilemmas. Teaching cyber-wisdom reasoning in the classroom could involve discussions of hypothetical and real-life online dilemmas, which aligns with the Jubilee Centre’s (2022) approach to teaching virtue reasoning. Research has shown that discussions of ethical dilemmas help students develop the ability to make moral decisions (Harrison et al., 2018; Hedayati-Mehdiabadi et al., 2020).

### Cyber-wisdom motivation

Cyber-wisdom motivation is a desire to act on virtues in relation to digital technologies and the online world. This concept aligns with Grossmann et al.’s (2020) ‘moral aspirations’ in their model of wisdom, and with Darnell et al.’s (2019) and Kristjánsson et al.’s (2021) blueprint component of *phronesis*. These models all focus on motivation to act on virtues and moral aspirations. Cyber-wisdom motivation specifically refers to the motivation to align one’s own behaviour with virtues in the online world, such as honesty and compassion. This means that users’ moral aspirations could include expectations for honest and compassionate interactions online, respectful discussions in online communities, and ethical design of the digital environment. Cyber-wisdom motivation can be taught through stories and discussions of exemplars and role models that encourage students to develop moral aspirations for online contexts. This approach aligns with the Jubilee Centre’s (2022) teaching of virtue identity and motivation, and with research on the benefits of this method for promoting character education (e.g. Zagzebski, 2017). For example, teachers could use examples of online activism against cyberbullying as a way to encourage virtue motivation.

### Cyber-wisdom self-reflection

Cyber-wisdom self-reflection is the ability to consider one’s own and others’ perspectives and emotions in the context of using digital technologies. This component builds on Ardelt’s (2004) concept of reflection, Grossmann et al.’s (2020) perspective-taking, and Darnell et al.’s (2019) and Kristjánsson et al.’s (2021) emotional regulation in their model of *phronesis*. These models all emphasize the importance of self-reflection for developing good character and regulating emotions. Cyber-wisdom self-reflection specifically focuses on the online world and the need to reflect on biases and emotions in dealing with moral dilemmas online, such as polarization and anger in response to online abuse. This aspect of cyber-wisdom can be taught through journaling and diary writing, which has been shown to encourage character development through self-reflection (Arthur et al., 2014). This aligns with the Jubilee Centre’s (2022) approach to teaching virtue emotions, and could involve asking students to reflect on the moral implications, biases, and emotions inherent in their own online experiences.

### Methodology

In this section we report on the participants for this study and on the methods and instruments employed to conduct an initial validation of the new measures developed for each of the four components of cyber-wisdom. The research objective for the study was to create and test out a new set of measures that could be used in future research in the field of cyber-wisdom education. With this objective in mind, the study sought to answer the following research question: can new measures of the components of cyber-wisdom be developed and preliminarily validated? To answer this question it was important to find establish theory-measurement congruence (i.e. model fit), internal consistency, and preliminary viability of a new set of measures. Validating measures in the field of character education is a complex and sophisticated process and therefore the expectation was that the research would not result in a set of fully validated and robust measures for cyber-wisdom education. Rather, the study was seen as providing solid foundation for additional advanced and sophisticated measure validation tests.

### Instruments and scales for the four components of cyber-wisdom education

The scales used for the study were informed by the Darnell et al. (2022) measure of *phronesis*. This decision was taken as the *phronesis* measure was designed to map onto the four-component model of *phronesis*, which in turn was an important inspiration for the cyber-wisdom education four-component model. As in Darnell et al. (2022), the initial survey, in the form of a questionnaire, was designed by the research team to measure aspects of each of the four theoretical components of cyber-wisdom education by drawing on and adapting existing measures (see below). After the initial survey had been developed, a pilot study was conducted with three schools and 100 pupils to test if the survey was fit for the purpose of the study. The primary purpose of this pilot was to determine the suitability of the questions with a particular focus on language comprehension, as well as to see whether the questions would likely elicit meaningful responses. The aim was not to explore the validity and reliability of the measure as this was a purpose of the pilot study described in the article. After the pilot, the survey was adapted in several ways. Some questions were removed whilst the language of others was adapted. These decisions were primarily made after a consideration of response rates, missing data, and any ceiling and floor effects based on the data distributions for each question. The following section describes each of the measurers.

#### Cyber-wisdom literacy

To assess pupils’ cyber-wisdom literacy (i.e. their understanding of the ways in which different virtues may apply to specific contexts that relate to the use of digital technologies), the same general approach adopted by Darnell et al. (2022), according to which the constitutive function of *phronesis* is crucial to understanding the ethical features of a given situation. Besides drawing on this dimension of *phronesis* but in ways that, unlike Darnell et al.’s (2022) model, place emphasis on the use of digital technologies, Thoma et al.’s (2013) adolescent intermediate concept measure (AD-ICM) was also used and adapted with a view to presenting pupils with a short story. As in the case of Thoma et al.’s (2013) instrument, the story was followed by a question that asked them to select and rank up to four of the virtues that were most relevant to the story. However, unlike Thoma et al.’s (2013) instrument, the story that was designed and used to measure cyber-wisdom literacy referred to a fictional situation relating specifically to an incident of online abuse. That is, pupils were presented with the story of a fictional character called Anna, who finds out that her friend Rachel has been sending nasty messages online to one of her classmates, Irene. Anna is asked by their teacher who might be responsible and does not know what to do. Pupils were asked to choose from a list of eight virtues – i.e. honesty, compassion, justice, integrity, loyalty, humility, respect, and courage. According to their relevance to the story, these eight virtues were first ranked by an expert panel of eight members, including academics and teachers with expertise in the field of character education. The virtues were ranked by the panel in the following order: (1) integrity, (2) compassion, (3) honesty, (4) courage, (5) respect, (6) loyalty, (7) justice, and (8) humility.

### Cyber-wisdom reasoning: (i) dimensions of wise reasoning; and (ii) moral engagement

Utilising the same story, pupils were then asked two questions designed to measure their cyber-wisdom reasoning, which refers to the ability to choose the best course of action in the context of using digital technologies, especially when one or two virtues clash depending on context. The first question was adapted from Brienza et al.’s (2018) Situated Wise Reasoning Scale (SWIS), which was also used to measure moral adjudication in Darnell et al. (2022). While the original scale includes 20 items (four items for each of five dimensions: recognising others’ perspectives, considering how different outcomes might unfold, intellectual humility, viewing an event from the vantage point of an outsider, consideration of compromise/conflict resolution), the items used were reduced to one item per dimension so as to ensure that the questionnaire did not take too long to complete, which was deemed essential for maximising response rates. What is more, the items used were reworded to match the story that pupils were presented with. This means that they referred to elements of moral reasoning that pertained specifically to the evaluation of a situation that, unlike those used within both Brienza et al.’s (2018) and Darnell et al.’s (2022) instruments, was characterised by online abuse and the use of digital technologies. With this in mind, the items included: “I would put myself in the shoes of the other people involved in the story (e.g. Rachel, Irene, Mr Smith)” (dimension 1: recognition of other perspectives); “I would look for different solutions as the situation unfolds (e.g. talking to my parents, talking to Mr Smith, talking to Rachel, talking to Irene)” (dimension 2: consideration of change and multiple ways a situation may unfold); “I would double check whether my opinion and the opinions of the other people involved in the story (e.g. Rachel, Irene, Mr Smith) are correct” (dimension 3: intellectual humility); “I would try to see the situation from the point of view of people not involved in the story (e.g. other students, parents, teachers)” (dimension 4: view of an event from the vantage point of an outsider); and “I would view it as very important that the situation is resolved (e.g. hoping that Rachel decides to apologise while Mr Smith and Irene’s parents decide not to suspend Rachel or call the police)” (dimension 5: consideration of compromise/conflict resolution). Pupils were asked to rate each item from 1 (“I strongly believe this is a bad choice”) to 5 (“I strongly believe this is a good choice”). Since all items described actions that Anna could take to resolve the situation, higher scores indicated higher cyber-wisdom reasoning.

Adapted from Thoma et al.’s (2013) AD-ICM, the question that followed asked pupils what Anna should do, in order to determine levels of moral engagement. Again, unlike the original measure, the items in response to this question were reduced to six and adapted so as to refer more specifically, in line with the story, to courses of action that may result from the experience of witnessing an incident of online abuse. Participants were asked to rate each item using a five-point scale ranging from “I strongly believe this is a bad choice” to “I strongly believe this is a good choice”. Pupils were presented with the following items: “Even though Anna doesn’t like the messages that Rachel has sent, Anna should say or do nothing and mind her own business”; “Anna should tell Mr Smith that it’s her best friend Rachel who has been sending nasty messages to Irene”; “Since Anna has never really liked Irene, she should support her best friend Rachel by also sending nasty messages to Irene”; “Anna should talk to Rachel first and see whether she will apologise to Irene or tell Mr Smith on her own, and should tell the truth if Rachel doesn’t”; “Even though Anna doesn’t like the messages that Rachel has sent to Irene, she should protect her best friend Rachel and be ready to lie in her defence, if necessary”; and “Anna should talk to her parents or to other close friends and seek their advice”. Items in response to this question were classified as either morally engaged actions (e.g. “Anna should talk to her parents or to other close friends and seek their advice”) or morally disengaged actions (e.g. “Since Anna has never really liked Irene, she should support her best friend Rachel by also sending nasty messages to Irene”), with the latter items being negatively scored before answers were summed.

### Cyber-wisdom motivation: (i) Ideal digital world; and (ii) Moral reasons

This component of cyber-wisdom, which refers to a desire to act with virtues in line with a vision of the digital world, was measured through two sets of questions. There is a lack of instruments in the literature that tap into one’s own moral aspirations not so much, as in the case of Darnell et al.’s (2022) instrument, in terms of the relevance of moral qualities to their sense of self, but in ways that may be underpinned by a broader vision of society and align with deontological, utilitarian or virtue ethical reasons. As a result, the sets of questions designed to measure this component of cyber-wisdom were newly developed for the purposes of this study. Of these, the first item was designed to tap into pupils’ visions of their ideal digital world and of the responsibilities of different actors (i). The second item was designed to capture pupils’ moral reasons (ii) behind the ways in which they use the Internet and social media. More specifically, the first item asked pupils to use a five-point scale ranging from “I really disagree with this statement” to “I really agree with this statement” to give their response to questions such as “In my ideal digital world, people are kinder and show more respect to each other online”. Pupils taking the programme were expected to be more likely to agree with the statements that were worded positively (e.g. “In my ideal digital world, Internet companies [e.g. Google, Facebook] act more promptly to solve problems such as misinformation and online abuse [e.g. bullying, trolling].”), and to disagree with the statements that were worded negatively (e.g. “In my ideal digital world, the government doesn’t have a responsibility to address problems such as misinformation and online abuse [e.g. bullying, trolling].”).

Meanwhile, the second question asked pupils to use a five-point scale ranging from “not important to me” to “extremely important to me” to rate nine items, categorised as deontological, virtue ethical, and utilitarian reasons for how they use digital technologies, with three items per category. In response to the question, which was worded as “How important is it to you that you…?”, examples of items included: “…follow your parents’ rules when using the Internet (e.g. by not communicating with strangers on social media)?” (deontological reason); “…are honest when communicating with others online (e.g. by not spreading misinformation)?” (virtue ethical reason); and “…think about whether what you do online might get you into trouble (e.g. posting inappropriate photos on social media)?” (utilitarian reason).

### Cyber-wisdom self-reflection

Finally, to measure pupils’ cyber-wisdom self-reflection (i.e. their ability to navigate, in the context of using digital technologies, their own perspectives and those of others as well as their own emotions and those of others), pupils were asked a question that was adapted from Davis’ (1983) Interpersonal Reactivity Index (IRI), which was used to measure moral emotion in Darnell et al. (2022). While the original measure incorporates four different dimensions, only two dimensions were deemed suitable for the purposes of measuring cyber-wisdom self-reflection. These dimensions included perspective taking (i.e. adoption of others’ viewpoints) and empathic concern (i.e. an individual’s feelings of compassion and concern for others), while the dimensions that were deliberately left out included fantasy (i.e. tendency to transpose one’s own emotions into those of a fictitious character) and personal distress (i.e. feelings of personal anxiety). The dimensions that were retained were chosen because they best reflected the features of *cyber-wisdom* self-reflection, which requires users to use digital technologies in ways that allow them to navigate both different perspectives and different emotions. Pupils were asked to rate a total of eight items (four per dimension) using a five-point scale ranging from “does not describe me very well” to “describes me very well”. Not only was the number of items used reduced from the original measure, but they were also reworded so as to relate to the use of digital technologies. Examples of items included “I always try to look at everybody’s side of a disagreement on social media before I take a position” (perspective taking), and “When I see someone being bullied on the Internet, I feel protective towards them” (emphatic concern). Pupils who were considered to be *high* in cyber-Wisdom self-reflection would be those who are more likely to agree with the statements that were worded positively (e.g. “I am often quite touched by the positive things that I see on the internet [e.g. users donating money to charities]”), and to disagree with the statements that were worded negatively (e.g. “If I’m sure I’m right about something, I don’t waste much time reading through the different arguments of other people in their Internet posts”), with the latter being reverse-scored.

### Research design

The sampling strategy that was adopted to recruit schools and participants to pilot the new measures was both purposive and based on convenience. Seven schools were recruited using contacts known to the research team. Schools were selected, as shown in Table 1, with a view to maximising heterogeneity in terms of geographical location. Pupils from the seven schools in years 9 and/or 10 completed the surveys either online (using Qualtrics) or in hard copy.

**Table 1:** Overview of Schools and Pupils Participating in the Feasibility Study

School	Area in England	No. of survey responses included in the analysis for RQ1	Questionnaire format
1	Southern England	316	Online
2	Southern England	120	Online
3	Northern England	47	Hard copy
4	Southern England	139	Hard copy
5	Midlands	402	Hard copy
6	Midlands	101	Hard copy
7	Northern England	206	Hard copy

Survey data from the seven schools (*N* = 1,331) was utilised to assess the internal reliability and general suitability of the new cyber-wisdom measure. This was done after the data was cleaned and prepared for analysis. The demographics of the participants who completed the measure can be seen in Table 2 (the split between the seven schools can be seen in Table 1).

**Table 2:** Participant Demographics and Time Spent Using the Internet Each Day

		*N* =	*N* %
Gender*	Female	707	55.8
	Male	531	41.9
	Other	30	2.4
Age	13	426	32.2
	14	690	52.2
	15	205	15.5
Time spent using the Internet each day	Little or no time	11	0.8
	About half an hour	22	1.7
	About 1 h	72	5.5
	About 2 h	155	11.7
	About 3 h	232	17.6
	About 4 h	260	19.7
	About 5 h	163	12.3
	More than 5 h	405	30.7

Note. *Total *N*s for Gender (*N* = 1268) and Age (*N* = 1321) are not the same due to missing data, so we also expressed Gender and Age proportionally as a percentage.

### Data analysis

Once the data outlined in Table 1 was cleaned and organised both on SPSS (i.e. the survey data collected via Qualtrics) and on an Excel spreadsheet (i.e. the survey data collected via hard copies), it was collated and analysed using SPSS (version 22) and STATA (version 16). In instances where over 50% of the data was missing on a survey it was removed from the analysis. When possible, the internal consistency of each component was assessed through Cronbach alpha tests. Then, to see how the items mapped into the theorised cyber-wisdom model, confirmatory factor analyses were performed.

### Ethical considerations

For each of the methods used in this study, ethical approval was granted by the University of Birmingham Ethics Committee. Adherence to ethical considerations was regarded as essential throughout the study, especially considering that this research was carried out with adolescents. Schools were informed about the study and a member of staff from each school was required to agree to the trial and give permission for their pupils to be involved. Letters were sent out to pupils and parents explaining the nature of the study, and parents were given the opportunity to opt their children out from being involved in the study.

### Findings

#### Cyber-wisdom literacy

A single measure was designed in an attempt to measure cyber-wisdom literacy. For its calculation, respondents’ answers were compared against the selection made by an expert panel. Each time a respondent agreed with the expert panel, they received a score of one. Considering this, the maximum score a student could get was four points. For this particular scale, the internal reliability could not be calculated because it was composed of four items scored on a binary scale, and so it violates Cronbach’s alpha test assumptions. In terms of response rates, 16.8% (224) of respondents marked fewer than four virtues and 4.8% (65) did not express any preference. Overall, scores appeared to be approximately normally distributed, showing no evidence of ceiling or floor effects and that scores may be amenable to change.

### Cyber-wisdom reasoning: (i) dimensions of wise reasoning; and (ii) Moral engagement

Two questions were used to measure cyber-wisdom reasoning. The first question is an adapted version of Brienza et al.’s (2018) Situated Wise Reasoning Scale (SWIS), and it is composed of five *Dimensions of Wise Reasoning* (intellectual humility; recognition of uncertainty and change; consideration of the broader context at hand; perspectives of others; integration of these perspectives or compromise). The second question, adapted from Thoma et al.’s (2013) AD-ICM, tapped into pupils’ levels of *Moral Engagement*.

**Fig. 1:**
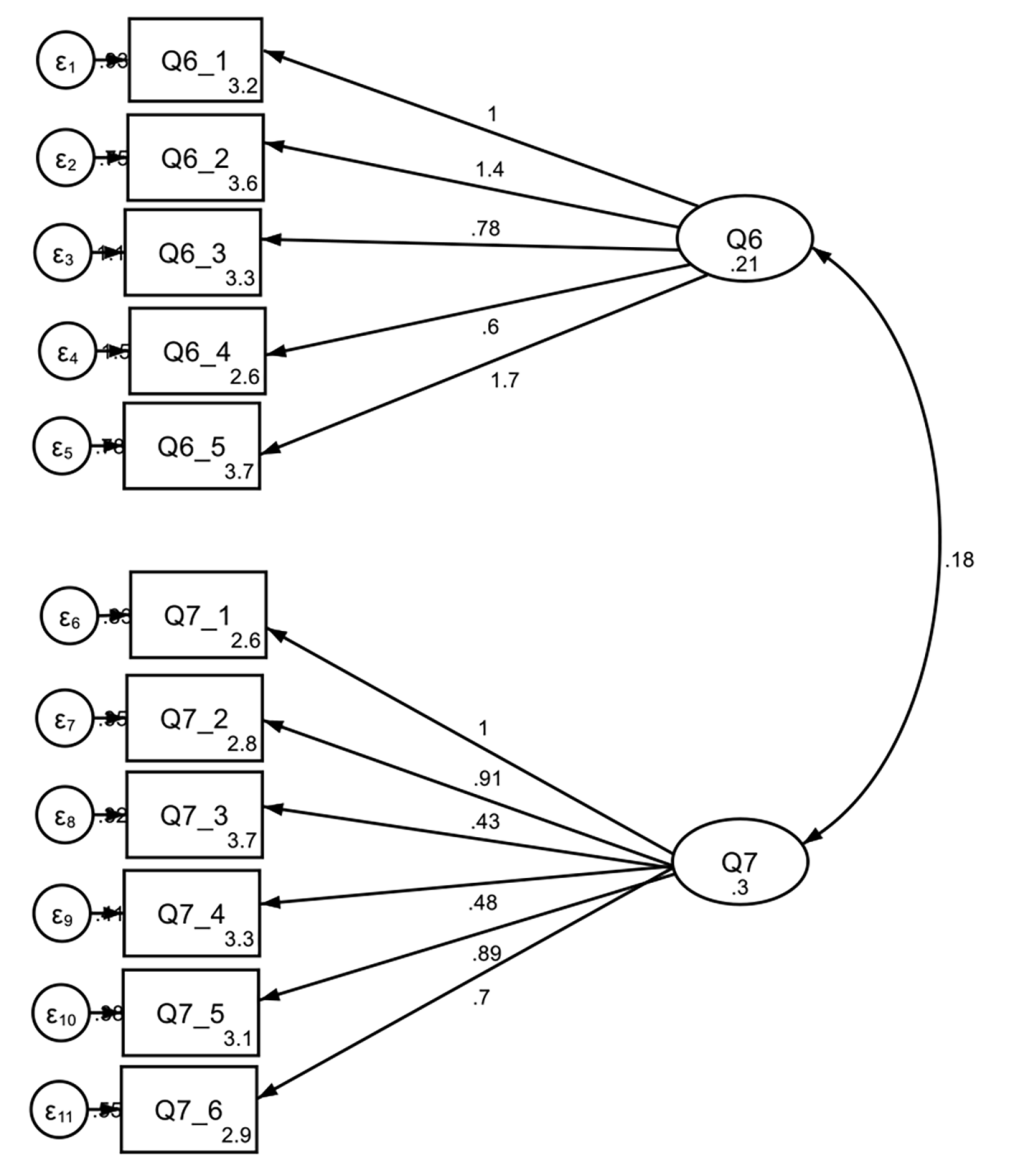
CFA for Cyber-Wisdom Reasoning; Moral Engagement and Dimensions Of Wise Reasoning

In terms of reliability, we can observe that the *Moral Engagement* question had a good Cronbach’s alpha of 0.70 and the *Dimensions of Wise Reasoning* question had a lower alpha of 0.58. The response rates were high in both measures with a non-response rate of 2.4% for *Moral Engagement* (33) and 5.9% (79) for the *Dimensions of Wise Reasoning* question. To test how the responses mapped onto the theoretical model, a confirmatory factor analysis was performed. The model (see Fig. 1) showed a reasonably high model fit (*RMSEA* = 0.058; *TLI* = 0.84; *CFI* = 0.88), given this preliminary validation stage. This suggests that the eleven items across the two questions do collectively measure two different aspects of cyber-wisdom reasoning. However, these items might be refined or reduced further to improve model fit with reference to modification indices.

### Cyber-wisdom motivation: (i) Ideal digital world; and (ii) Moral reasons

**Fig. 2:**
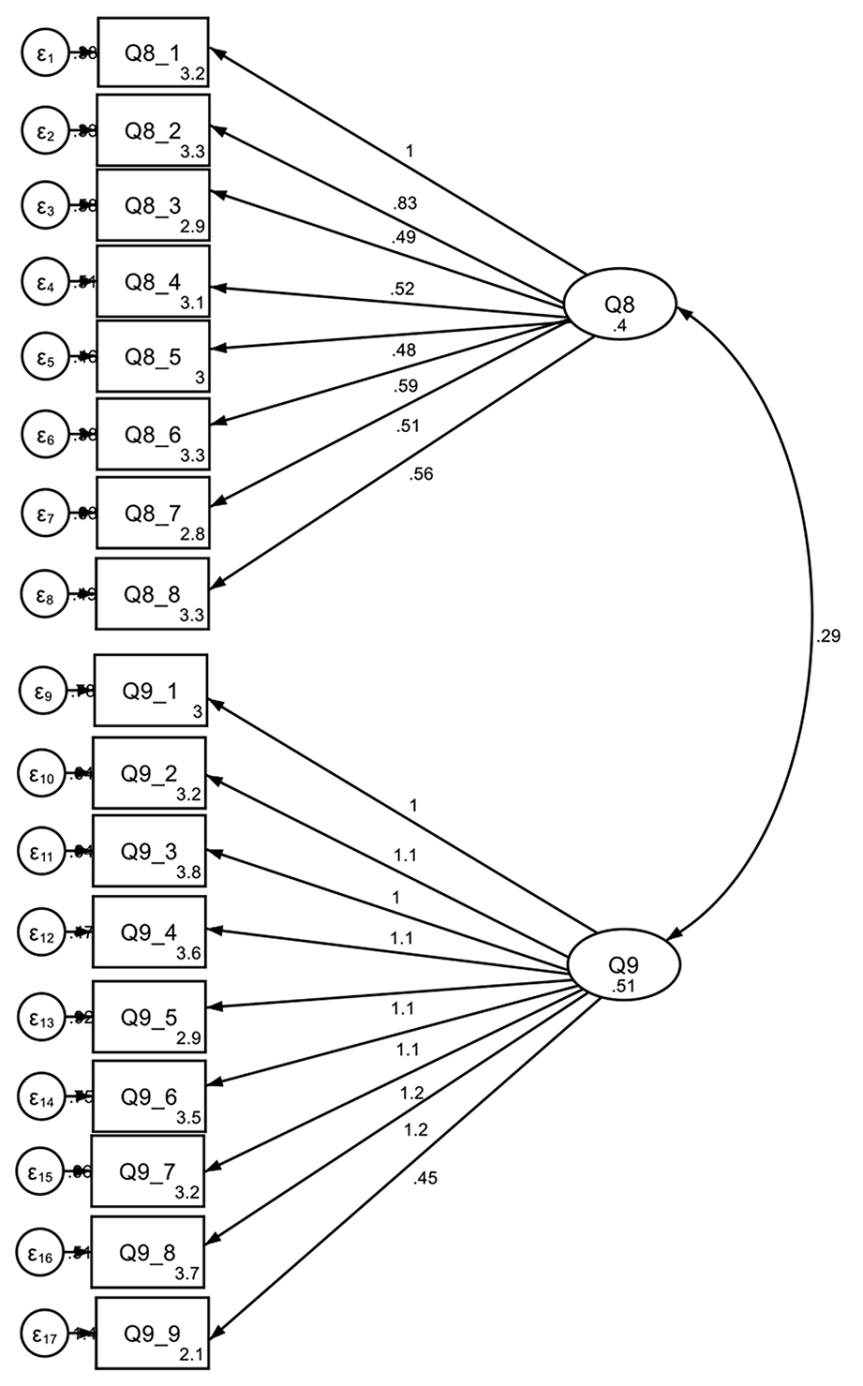
CFA for Cyber-Wisdom Motivation; Ideal Digital World and Moral Theory

This component of cyber-wisdom, which refers to a desire to act with virtues in line with a vision of the digital world, was measured through two questions. Of these, the first question was designed to tap into pupils’ visions of their ideal digital world and the second to determine which moral reasons motivated their actions in ways that resonate with different moral theories. Following the previous trend, the response rates showed a high level of compliance (non-response rate *Ideal Digital World*: 5.4% [73]; *Moral Reasons*: 1.9% [26]). The reliability of each scale was satisfactory: the Cronbach alpha was 0.75 for *Ideal Digital World* and 0.80 for *Moral Reasons*. Confirmatory factor analysis (see Fig. 2) again showed a promising model fit (*RMSEA* = 0.07; *TLI* = 0.85; *CFI* = 0.87) for this preliminary validation, suggesting that the seventeen items do collectively measure two aspects of cyber-wisdom motivation. Once again, these items could be reduced further to improve model fit based on an examination of the modification indices.

### Cyber-wisdom self-reflection

To measure pupils’ cyber-wisdom self-reflection (i.e. their ability to navigate, in the context of using digital technologies, their own perspectives and those of others, as well as their own emotions and those of others), participants were asked a question that was adapted from Davis’ (1983) Interpersonal Reactivity Index (IRI). Similar to the other scales, the response rate was high, with only 4.3% (58) of the sample not answering the full eight items. This scale showed good internal consistency, with a Cronbach’s alpha of 0.69. As a single factor scale (see Appendix 1), an excellent model fit was found (*RMSEA* = 0.06; *TLI* = 0.92; *CFI* = 0.94).

### Cyber-wisdom – overall measure

We undertook a confirmatory factor analysis to test how well the theorised model fitted the data overall. The virtue literacy component (Q5) was left out due to its measurement limitations discussed above. When the remaining questions were entered, the resulting model had a promising degree of model fit (*RMSEA* = 0.05; *TLI* = 0.85; *CFI* = 0.86) given the preliminary nature of this study, suggesting that these theoretically derived components may fit together empirically within the same model, pending future refinement of the measure. A summary of the overall model fit, as well as the sub-scale model fit and internal reliability statistics, can be found in Table 3.

**Table 3:** Model fit indices for Cyber-Wisdom Reasoning, Motivation and Self-Reflection

		α	TLI	CFI	RMSEA
Overall Measure			0.85	0.86	0.05
Cyber-Wisdom Reasoning	Dimensions of Wise Reasoning	0.58	0.84	0.88	0.06
	Moral Engagement	0.70			
Cyber-Wisdom Motivation	Ideal Digital World	0.75	0.85	0.87	0.07
	Moral Reasons	0.80			
Cyber-Wisdom Self-Reflection		0.69	0.92	0.94	0.06

## Discussion

Cyber-wisdom education is gaining increasing attention from researchers and practitioners (Harrison, 2021a,  Arthur et al., 2022). Whilst the theory and practice of cyber-wisdom education is established, to date there has been no attempt to investigate how the meta-virtue of cyber-wisdom might be measured. This is a lacuna as it limits future research in the area, including proximal evaluations of cyber-wisdom interventions. This study was an attempt to construct and undertake preliminary validation of the measures for the four-components of cyber-wisdom. Promisingly, the findings show that the four-part conceptualisation of cyber-wisdom can build on previous research on the meta-virtue of *phronesis* (see Darnell et al., 2019) and wisdom more broadly (see, Grossman et al., 2020), providing a useful theoretical foundation for the development of new measures that take account of the specificity of the digital age.

The cyber-wisdom literacy measure was adapted from Thoma et al.’s (2013) adolescent intermediate concept measure (AD-ICM). The high response rate suggests the dilemma that focused specifically on online abuse was understood by the participants. The adapted AD-ICM appears to be a useful face-valid measure of what it attempts to index. However, due to the small number of items in the adapted measure, it may not capture the full variation of cyber-wisdom literacy across participants. In the future, it would be good to include additional questions that seek to tap further into the component of cyber-wisdom literacy to more fully capture interpersonal variation in cyber-wisdom literacy.

The two-part cyber-wisdom reasoning measure was adapted from Brienza et al.’s (2018) Situated Wise Reasoning Scale (SWIS) and Thoma et al.’s (2013) AD-ICM. The adapted scales were considered to be promising measures separately. In addition, a confirmatory factor analysis showed that they also had a generally acceptable two-factor model fit. This is a promising finding given the difficulty of measuring a concept wherein the ‘correct’ answer (i.e. what it means to reason wisely) is highly subjective. Given its properties, this two-part measure of cyber-wisdom reasoning could be used in future research.

The two-question cyber-wisdom motivation measure was not adapted from any existing measures. Given this, it was positive to note that after analysis it was considered a suitable fit to the theorised model, given its stage of development and novelty. The second part of the model, which built on previous Jubilee Centre’s studies that assess responses against three prominent moral theories (see, for example, Arthur & Earl, 2020) did provide some interesting results, similar to findings reported in the *A Cyber-wisdom Approach to Digital Citizenship Education* (Harrison et al., 2022a) study. The self-reporting of motivation might be considered to be problematic, and so either (i) alternative conceptualisations of motivation might be desirable in future studies, or (ii) self-reported cyber-wisdom’s criterion validity could be established by predicting actual behaviour. The latter reflects an alternative conceptualisation in which motivation can only be inferred from action. For example, applied behavioural scientists use functional behavioural assessments to track the consequences of behaviours and subsequently infer the motivations behind them (see Gresham et al., 2001).

The fourth component, cyber-wisdom reflection, was measured through one question that was adapted from Davis’ (1983) Interpersonal Reactivity Index (IRI). This measure showed good internal consistency and excellent model fit as a single factor scale, and is worthy of consideration for use in future studies. The IRI was previously used to measure moral emotion in Darnell et al.’s (2022) preliminary validation of the *phronesis* inventory.

The component measures of cyber-wisdom generally showed promising psychometric properties, ratifying the decision to base the construction of the scales on Darnell et al.’s (2022) measure of *phronesis*. The measures have been *preliminarily* validated in certain respects here, but may be refined in future research studies to improve their psychometric properties (especially model fit, convergent validity, and test-retest reliability) and to suit researchers’ needs (e.g. to make it faster to administer and score, standardisation etc.). Multiple versions of cyber-wisdom tests may be desirable depending on the aims of the studies in which they are used. For example, measures such as the AD-ICM and functional behavioural assessments can take a long time to administer and score, which presents additional challenges when they are used in larger scale randomised trials. Tests of constructs such as cyber-wisdom literacy and reasoning could be more scalable if objectively ‘correct’ answers were determined in advance, making the scoring process faster and more transparent. The use of experiential and other types of measures to enhance these self-report measures should also be considered. Finally, it would be interesting to explore how measures might be integrated into the online apps that are popular among adolescents to improve user acceptability and scalability from a research perspective.

### Limitations

The present study has several limitations, which are commonly acknowledged in the field of character-education research. The study used a theoretically-derived model that included a wide range of measures, each with complex scoring formats. As a result, the theory-to-model fit scores were relatively high, but could be improved in future studies. One potential way to do this would be to eliminate some items based on modification indices, or to use different measurement instruments to assess the same four theoretically-derived cyber-wisdom sub-factors. Another limitation of the study is its non-probabilistic sampling strategy. The schools included in the sample were selected purposively and based on convenience, which means that the sample is likely to be biased. This bias is compounded by the fact that the recruitment of schools relied heavily on gatekeepers, who were mostly teachers. As a result, only those who were initially attracted to the study participated, creating a ‘self-selection’ sampling bias. Due to these limitations, the findings of the study cannot yet be reliably generalised to the broader population. Additionally, all of the measures used in the study were based on self-reporting, which can lead to issues of self-deception and social desirability (Weber & Cook, 1972). Self-deception occurs when participants provide inaccurate responses in accordance with how they would like to perceive themselves, rather than how they actually are. Social desirability, on the other hand, occurs when participants are motivated to present themselves in a favourable light. These concerns can be mitigated in future studies by assessing convergent validity with real-world behaviour. This would involve showing that what people say in a self-report measure corresponds to what they do in the real world.

## Conclusion

Cyber-wisdom is defined in this article as the ability to do the right thing at the right time, when using digital technologies. Drawing on virtue ethical moral theory, it is conceptualised as a virtue that helps users to maximise online opportunities and minimise online risks. The task of educating cyber-wisdom in children and adolescents relies on joint efforts from multiple stakeholders, including parents, teachers, policymakers, and technology companies. Despite the virtue being valued by parents and adolescents, there is still much to learn about how it might be measured. This paper presents promising findings from a study that sought to provide a preliminary validation of a four-component model of cyber-wisdom that could inform educational practice. Given the novelty of the research, the findings need to be treated with a degree of caution until the measure can be refined further. Despite this cautionary note, the study has provided preliminary evidence that the four theoretically-derived components of cyber-wisdom (literacy, reasoning, motivation, and reflection) may fit within a psychometric model. Further research is required to adapt the measures and provide further validation of their psychometric properties (see Harrison et al., 2022b).

## Data Availability

The datasets generated during and/or analysed during the current study are available from the corresponding author on reasonable request.

## Footnotes

1 https://www.commonsensemedia.org.
